# Effect of perceived stress on sleep quality in Chinese graduate students: the chain mediating role of anxiety and sleep procrastination

**DOI:** 10.3389/fpsyg.2026.1781661

**Published:** 2026-06-02

**Authors:** Xiaomeng Hu, Zhehao Liu, Chunyan Sui, Zihang Xu, Xin Miao, Yunge Zheng, Jiawei Zhou, Tianyi Bu, Yuecui Kan, Kexin Qiao, Xuan Liu, Yanjie Yang, Zhengxue Qiao

**Affiliations:** 1 Psychology and Health Management Center, Harbin Medical University, Harbin, China; 2 The Forth People’s Hospital of Chengdu, Chengdu, China

**Keywords:** anxiety, chain mediating effect, perceived stress, sleep procrastination, sleep quality

## Abstract

**Background:**

In recent years, with the acceleration of the pace of modern life and the increase of life pressure, the prevalence of poor sleep quality has risen substantially among young people. Insufficient sleep and insomnia have emerged as a serious public issue in modern society. This study aimed to investigate the effect of perceived stress on sleep quality, with particular focus on the mediating roles of anxiety and sleep procrastination in the stress-sleep quality relationship.

**Methods:**

A total of 2,486 Chinese graduate students participated in the questionnaire survey, which used the Perceived Stress Scale, the Bedtime Procrastination Scale, the Pittsburgh sleep quality index, and the Self-rating Anxiety Scale. Descriptive statistics, correlation analysis, and the bootstrap method were utilized for data analysis.

**Results:**

The findings revealed a 37.8% prevalence of sleep disturbances among the participants. Notably, the mediating effects of anxiety and sleep procrastination in the association between perceived stress and sleep quality were statistically significant (*p* < 0.05).

**Conclusion:**

The results showed that higher perceived stress was positively associated with greater anxiety, which further contributed to sleep procrastination, and ultimately correlated with poorer sleep quality.

## 1 Background

Sleep constitutes a fundamental aspect of human life, representing approximately one-third of an individual’s lifespan. In recent years, the accelerating pace of modern life has led to significant shifts in living conditions, learning environments, and work patterns. Consequently, the prevalence of poor sleep quality has risen substantially. Insufficient sleep and insomnia have emerged as a serious public issue in modern society. A health survey by Kernan et al. (2011) involving 1,355 American graduate students found that 51.7% had difficulty sleeping. Allen et al. (2021) completed an online survey of a sample of 2,683 master’s, Ph. D., and professional graduate students from two large public universities. The study found that participants slept an average of 6.4 h per night, with 38% reporting poor sleep quality (Kernan et al., 2011). Sleep disorders exert serious impacts on graduate students’ education, daily lives, and physical and mental health (Townsend, 2010). Therefore, investigating the factors influencing sleep quality in this population is of great importance.

Poor sleep quality is common among professional graduate students due to stress related to academic and work demands. Some studies have shown that, compared with undergraduate students, graduate students are more likely to report experiencing “high pressure” or “above-average pressure” (Wyatt and Oswalt, 2013). According to the health survey of graduate students by Kernan et al. (2011), 75.4% reported feeling stressed. A study found that the sleep quality of college students is significantly negatively affected by perceived stress (Li et al., 2019). Furthermore, a study of 196 master’s students in the United States found that most participants exhibited impaired sleep quality, which was significantly associated with higher perceived stress levels, poorer sleep hygiene and environments, and a greater number of lifetime traumatic events (Lee et al., 2022). A longitudinal outcome study (Eliasson et al., 2010) of changes in perceived stress and their correlation with sleep quality found that a decrease in perceived stress was significantly associated with improved sleep quality. However, the specific psychological mechanisms underlying how perceived stress affects sleep quality in graduate students remain unclear, particularly with regards to the sequential mediating roles of emotional and behavioral factors in this relationship.

Perceived stress, defined as the subjective appraisal of external stress as threatening or unmanageable, consists of two distinct dimensions: crisis perception (the tendency to perceive daily events as stressful and threatening) and coping ability perception (the subjective evaluation of stressors). This two-dimensional structure enables a more nuanced understanding of how stress appraisal impacts psychological and behavioral outcomes. Extant studies have consistently identified a robust association between perceived stress and sleep quality, and this relationship can be explained from both physiological and psychological perspectives. Physiologically, perceived stress activates the hypothalamic–pituitary–adrenal (HPA) axis, leading to elevated cortisol levels that disrupt the circadian rhythm and sleep architecture (Van Reeth et al., 2000), while psychologically, perceived stress triggers repetitive negative thinking and emotional arousal, both of which interfere with sleep initiation and maintenance (Harvey, 2002). However, the direct effect of perceived stress on sleep quality only partially explains this relationship, highlighting the urgent need to explore intermediate variables that transmit this effect.

Anxiety is an emotional state characterized by subjective feelings of unpleasantness and inner turbulence, encompassing fear of anticipated events. It may play a crucial role in the impact of perceived stress on sleep quality. Researchers have found that individuals with higher levels of anxiety traits and negative coping strategies may experience further increases in perceived stress levels when faced with psycho-social pressure (Garbóczy et al., 2021). In addition, studies have shown a close relationship between perceived stress and anxiety (Dong et al., 2023). The relationship between anxiety and sleep quality has been explored to a considerable extent in existing literature. A study found that there is a bidirectional relationship between anxiety and sleep quality (Chellappa and Aeschbach, 2022). This research indicated that approximately 50% of patients with anxiety have sleep disorders, particularly insomnia, and that sleep deprivation can trigger or further exacerbate anxiety. Anxiety has negative impacts on daytime alertness and overall sleep quality (Oh et al., 2019). Psychologically, anxiety elevates pre-sleep cognitive arousal, disrupting sleep initiation and maintenance, which may be the key mechanism linking perceived stress to poor sleep quality via anxiety.

While anxiety is potentially a key mediating factor, it is also necessary to explore how sleep procrastination interacts with perceived stress to affect sleep quality. The concept of sleep procrastination was introduced by Kroese et al. (2014) in the field of health behavior procrastination, referring to the phenomenon of “failing to go to bed at a predetermined time despite the absence of external barriers.” Sleep procrastination was an independent predictor of poor sleep quality, with students exhibiting more sleep procrastination behaviors having approximately 2.5 times higher risk of reporting poor sleep quality (Ma et al., 2022). A meta-analysis by Hill et al. (2022) also revealed that sleep procrastination is a unique factor that contributes to the severity of poor sleep quality and is negatively correlated with sleep quality. From the perspective of the self-regulatory resource theory (Muraven and Baumeister, 2000), self-regulatory resources are limited, and perceived stress and subsequent anxiety consume a large amount of self-regulatory resources of individuals (Garbóczy et al., 2021), which are resources that are necessary for adhering to planned behaviors such as maintaining a regular bedtime. When self-regulatory resources are depleted, individuals are more likely to engage in immediate gratification behaviors and delay sleep, leading to sleep procrastination (Muraven and Baumeister, 2000). In addition, sleep procrastination may serve as an avoidant coping strategy for individuals with high anxiety, who use delayed bedtime to escape from stress-related negative emotions (Hill et al., 2022). This suggests a potential sequential relationship; anxiety, as an emotional response to perceived stress, may further trigger sleep procrastination, a behavioral outcome of self-regulatory failure.

Harvey’s cognitive model of insomnia (Harvey, 2002) suggests that excessive and uncontrollable worries about life stressors before bedtime can lead to emotional arousal, resulting in anxiety that can cause cognitive biases toward stressful events and increased attention to the threats posed by these stressors. Consequently, individuals may evaluate stress events in a distorted manner, subjectively perceiving a decline in sleep quality. According to the self-regulatory resource theory (Muraven and Baumeister, 2000), self-regulatory resources are limited, and engaging in resource-consuming activities, such as emotion regulation and thought control, can impair self-control. When individuals perceive higher levels of stress, they may expend excessive self-regulatory resources during the day, leaving insufficient reserves to resist staying up late at night. This failure in self-regulation can subsequently lead to sleep procrastination. Existing domestic and international studies have demonstrated the individual mediating roles of anxiety and sleep procrastination in the perceived stress-sleep quality relationship, but few have explored their combined serial mediating mechanism (Ma et al., 2022; Hill et al., 2022). The existing literature lacks a clear theoretical justification for the serial pathway of perceived stress → anxiety → sleep procrastination → poor sleep quality, and the two dimensions of perceived stress have not been differentiated in exploring these mediating mechanisms. This study therefore addressed these research gaps by testing a serial mediation model, which was the primary novelty and contribution of the present work. By clarifying the sequential transmission mechanism of emotional and behavioral factors between perceived stress and sleep quality among Chinese graduate students, and by exploring the differential effects of the two dimensions of perceived stress on this serial mediation model, this study aimed to provide a more comprehensive theoretical explanation for sleep disturbances in this population, and suggested targeted practical guidance for improving sleep quality and mental health.

Based on the above theoretical analysis and existing empirical evidence, the following hypotheses are proposed.

*H1*: Perceived stress is significantly positively associated with poor sleep quality in Chinese graduate students (direct effect).

*H2*: Anxiety mediates the relationship between perceived stress and sleep quality (perceived stress → anxiety → sleep quality), such that higher perceived stress is associated with elevated anxiety, which in turn is related to poorer sleep quality.

*H3*: Sleep procrastination mediates the relationship between perceived stress and sleep quality (perceived stress → sleep procrastination → sleep quality), such that higher perceived stress is associated with more severe sleep procrastination, which in turn is related to poorer sleep quality.

*H4*: Anxiety and sleep procrastination play a serial mediating role in the relationship between perceived stress and sleep quality (perceived stress → anxiety → sleep procrastination → sleep quality), such that higher perceived stress is associated with elevated anxiety, which further increases sleep procrastination, ultimately being related to poorer sleep quality.

*H5*: The two dimensions of perceived stress (crisis perception, coping ability perception) show differential effects on the serial mediation model.

## 2 Materials and methods

### 2.1 Participants

This study adopted a convenience sampling approach, recruiting graduate students from three universities in Harbin (Harbin Medical University, Harbin University of Science and Technology, and Heilongjiang University) through course platforms and social media from September to October 2024. An electronic survey was administered via the online platform ‘Questionnaire Star’. A total of 2,863 questionnaires were collected. After excluding invalid questionnaires, 2,486 valid responses were retained, yielding an effective response rate of 86.83%. The present study was approved by the Ethics Committee of Harbin Medical University (HMUIRB20200002). All participants provided informed consent prior to completing the questionnaire.

Invalid questionnaire exclusion criteria were operationalized as follows: (1) uniform or regular response bias (all items with the same answer or cyclic scoring such as 1–2–3-4), (2) logically inconsistent responses (contradictory answers to relevant items), and (3) incomplete answers (missing responses for more than one-third of the items on the core scales).

### 2.2 Measurement

#### 2.2.1 Basic information questionnaire

Developed by the researchers, the questionnaire mainly included items such as age, sex, subject major (Medicine, Literature and History, or Science and Engineering), educational level (Masters level or above), and hometown. Lifestyle variables included frequency of smoking, frequency of alcohol consumption, frequency of coffee drinking, frequency of exercise before bedtime, frequency of breakfast intake and dormitory environment (rated as good/fair/poor). These lifestyle variables were included as covariates in the subsequent analyses because prior research has confirmed significant associations with perceived stress, anxiety and sleep quality (Rassolnia and Nobari, 2024), and their role as possible confounding factors in the stress-sleep relationship.

#### 2.2.2 Perceived stress (perceived stress scale, PSS-10)

The Perceived Stress Scale (PSS) was originally developed by Cohen and Williamson (1988) and later revised into a Chinese version by Yuan and Lin (2009). For the current study, the Cronbach’s *α* coefficient of the scale was 0.768. Scoring criteria: the scale consists of 10 items across two distinct dimensions, namely crisis perception (six items: 1, 2, 3, 6, 9, 10) and coping ability perception (four items: 4, 5, 7, 8) (Yuan and Lin, 2009). The crisis perception dimension includes six negatively worded items (items 1, 2, 3, 6, 9, 10), while the coping ability perception dimension comprises four positively worded items (items 4, 5, 7, 8). A Likert five-point scale was used for scoring (0 = never, 1 = seldom, 2 = sometimes, 3 = often, 4 = always). Items 4, 5, 7, and 8 were reverse-scored; thus higher scores on these items reflected lower levels of the construct. After reverse scoring, higher total scores across all items indicated greater perceived stress.

#### 2.2.3 Sleep procrastination (bedtime procrastination scale, BPS)

The Bedtime Procrastination Scale was developed by Kroese et al. (2014) and translated into a Chinese version by Ma et al. (2022). The Cronbach’s alpha coefficient of the scale in this study is 0.829. Scoring criteria: the scale consists of nine items measured on a five-point Likert scale ranging from 1 (“almost never”) to 5 (“almost always”). Among these items, items 2, 3, 7, and 9 are reverse-scored. The average score of the nine items is typically used as the overall scale score. The score range is from 1 to 5, with higher scores indicating more severe sleep procrastination.

#### 2.2.4 Sleep quality (Pittsburgh sleep quality index, PSQI)

The Pittsburgh Sleep Quality Index (PSQI) was developed by Buysse and other psychiatrists at the University of Pittsburgh in the United States, and it is suitable for assessing sleep quality in the general population. Liu et al. (1996) translated the scale and demonstrated good reliability and validity among college students. Scoring criteria were: the PSQI assessed participants’ sleep quality over the past month and was comprised of 19 self-rated items and five observer-rated items. Items excluded from scoring for practical and methodological reasons: the 18th self-rated item (an open-ended question about the impact of sleep problems on daily life, which is difficult to quantify) and all five observer-rated items (which cannot be collected in an online survey without objective raters). The remaining 18 self-rated items were grouped into seven components: sleep quality, sleep latency, sleep duration, sleep efficiency, sleep disturbances, use of sleeping medication, and daytime dysfunction. Each component was scored from 0 to 3, and the total score was the sum of the seven components (range: 0–21). A higher total score indicated poorer sleep quality. According to Liu et al. (1996), total scores greater than 7 indicated poor sleep quality, scores less than 3 indicated good sleep quality, and scores of 3–7 indicated average sleep quality. The Cronbach’s alpha coefficient of the scale in this study is 0.88.

#### 2.2.5 Anxiety (self-rating anxiety scale, SAS)

The Self-rating Anxiety Scale (SAS) is a clinical measurement tool developed by Zung (1971) for assessing subjective symptoms of anxiety in patients. The Cronbach’s alpha coefficient of the scale in this study is 0.867. The SAS consisted of 20 items, of which 15 were negatively worded and scored from 1 to 4. The remaining five items (items 5, 9, 13, 17, 19) marked with an asterisk (*) were positively worded and scored in reverse from 4 to 1. The primary statistical indicator of the SAS was the total score. To obtain the raw score, the scores of all 20 items were summed. The standard score was then calculated by multiplying the raw score by 1.25 and rounding-up. The cut-off score for anxiety assessment was 50. Scores ranging from 50 to 59 indicated mild anxiety, 60 to 69 moderate anxiety, and scores greater than 70 indicated severe anxiety (Zung, 1971).

### 2.3 Serial mediation analysis

This study used the PROCESS macro program (Model 6) in SPSS 26.0 to construct the serial mediation model, with perceived stress as the independent variable, sleep quality as the dependent variable, anxiety and sleep procrastination as serial mediators, and age, sex, major category, educational level, and lifestyle variables as covariates.

The nonparametric percentile bootstrap method with 5,000 resamples was used to test the significance of mediating effects, with a 95% confidence interval (95% CI). A mediating effect was considered statistically significant if the 95% CI did not contain 0.

The analysis procedure was as follows: (1) test the direct effect of perceived stress on sleep quality (H1), (2) test the simple mediating effects of anxiety (H2) and sleep procrastination (H3) separately, (3) test the full serial mediation model of perceived stress → sleep procrastination → sleep quality (H4), with the 95% confidence interval (CI) estimated, and (4) analyze the differential effects of the two dimensions of perceived stress on the serial mediation model (H5).

## 3 Results

### 3.1 Characteristics of the participants

A total of 2,486 students were involved in this study. Of these, 904 (36.4%) were male, with an average age of 24.91 ± 3.43 years. The distribution of students by academic qualifications showed that the majority (1,864, 75%) were masters level students, followed by doctoral students (622, 25%). Of the total number of respondents, 2,306 (92.76%) were in the medical profession. The participant characteristics are presented in Table 1.

**Table 1:** Participant demographics (*n* = 2,486).

Variables	Mean ± SD/*N* (percentage)
Age, mean ± SD	24.91 ± 3.43 years
Sex, *N* (%)	
Female	1,582 (63.64%)
Male	904 (36.36%)
Stage, *N* (%)	
Masters degree	1,864 (74.97%)
Doctorate degree	622 (25.03%)
Major, *N* (%)	
Medicine	2,306 (92.76%)
Literature and History	19 (0.76%)
Science and Engineering	161 (6.48%)

### 3.2 Descriptive statistics and correlations

The results of this study revealed that the prevalence of sleep disorders among graduate students in China was 37.8%. The descriptive statistics and correlations of the study are presented in Table 2. The correlation results indicated a significant positive correlation among the four variables of perceived stress, anxiety, sleep procrastination, and sleep quality.

**Table 2:** Descriptive statistics and correlations (*n* = 2,486).

Variables	Perceived stress	Anxiety	Sleep procrastination	Sleep quality
Perceived stress	1			
Anxiety	0.711^**^	1		
Sleep procrastination	0.377^**^	0.324^**^	1	
Sleep quality	0.464^**^	0.553^**^	0.422^**^	1
*M*	14.58	42.24	2.92	5.71
SD	6.130	10.445	0.777	3.326

^**^p < 0.01.

### 3.3 The chain mediating model

This study used Model 6 of the Hayes PROCESS macro to test for chained mediation; the results are presented in Table 3. The regression model with anxiety as the dependent variable showed that perceived stress had a significant positive predictive effect on anxiety (*β* = 0.711, *p* < 0.001, 95% CI = [1.165, 1.259]); The model with sleep procrastination as the outcome variable shows that perceived stress (*β* = 0.296, *p* < 0.001, 95% CI = [0.031, 0.044]) and anxiety (*β* = 0.113, *p* < 0.001, 95% CI = [0.005, 0.012]) both had significant positive predictive effects on sleep procrastination; The model with sleep quality as the outcome variable showed that perceived stress (*β* = 0.065, *p* < 0.01, 95% CI = [0.011, 0.060]) and anxiety (*β* = 0.423, *p* < 0.001, 95% CI = [0.120, 0.149]), and sleep procrastination (*β* = 0.26, *p* < 0.001, 95% CI = [0.970, 1.259]) all had significant positive predictive effects on sleep quality. The 95% confidence intervals for all paths do not include 0, indicating robust effects and suggesting that anxiety and sleep procrastination play significant parallel and chained mediating roles between perceived stress and sleep quality.

**Table 3:** Test of mediating effect (*n* = 2,486).

Outcome	Predictor	*B*	SE	*t*	*β*	95%CI
M1	X	1.212	0.024	50.42	0.711***	[1.165, 1.259]
M2	X	0.038	0.003	11.237	0.296***	[0.031, 0.044]
	M1	0.008	0.002	4.298	0.113***	[0.005, 0.012]
Y	X	0.035	0.013	2.806	0.065**	[0.011, 0.060]
	M1	0.135	0.007	18.63	0.423***	[0.120, 0.149]
	M2	1.114	0.074	15.117	0.260***	[0.970, 1.259]

^***^p < 0.001, ^**^p < 0.01.

X = perceived stress, M1 = anxiety, M2 = sleep procrastination, Y = sleep quality.

**Figure 1:**
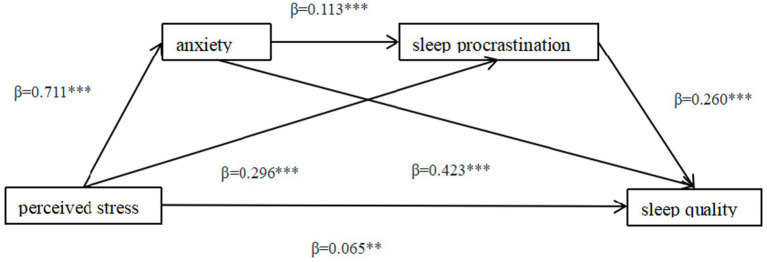
Diagram of the chain mediation model. ^***^p < 0.001, ^**^p < 0.01.

To test the significance of indirect effects in the serial mediation model, a bias-corrected bootstrap analysis with 5,000 resamples, was conducted. Indirect effects were deemed significant if their 95% CIs did not include 0. As shown in Table 4 and Figure 1, the 95% CIs for all indirect paths did not contain 0, indicating significant mediation effects.

**Table 4:** The mediation effect analysis (*n* = 2,486).

Paths	Effect	95%confidence interval
X → M1 → Y	0.163	0.141–0.184
X → M2 → Y	0.042	0.032–0.052
X → M1 → M2 → Y	0.011	0.006–0.017
Total indirect effect	0.216	–

*N* = 2,488. Level of confidence for all confidence intervals: 95%.

X = perceived stress, M1 = anxiety, M2 = sleep procrastination, Y = sleep quality.

The results revealed that in Model 1, anxiety significantly mediated the association between perceived stress and sleep quality [indirect effect = 0.163, 95% CI: (0.141, 0.184)]. In Model 2, sleep procrastination also emerged as a significant mediator in this relationship [indirect effect = 0.042, 95% CI: (0.032, 0.052)]. Building on these preliminary findings, we subsequently tested the full serial mediation model (Perceived Stress → Anxiety → Sleep Procrastination → Sleep Quality). The results indicated that anxiety not only exerted a direct effect on sleep quality but also exerted an indirect influence via its impact on sleep procrastination, forming a significant chained pathway [indirect effect = 0.011, 95% CI: (0.006, 0.017)]. In summary, all three models yielded significant mediation effects. The serial mediation model further elucidated a more complex dynamic mechanism underlying the relationships among these variables, thereby supporting its validity as the final theoretical framework (Figure 1).

Based on the results of the mediating effect analysis using the total score of the dependent variable, and in order to analyze the independent effects of each dimension within the dependent variable in greater depth—thereby avoiding the risk of relying solely on the total score, which may obscure differences in the effects between dimensions—this study further divides the dependent variable into two dimensions. Mediating models are constructed and tested separately for each dimension to examine their specific effects within the overall mediating pathway.

**Figure 2:**
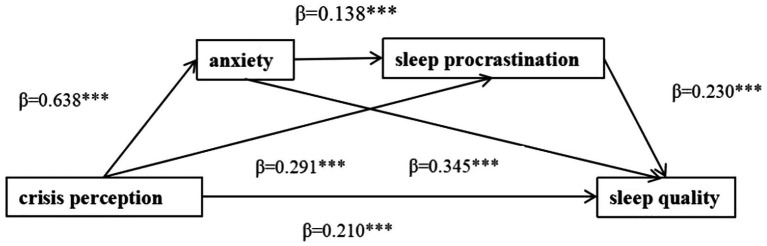
Diagram of the chain mediation model. ^***^p < 0.001.

Bootstrap results indicated that all hypothesized paths were significant. Crisis perception had a significant direct effect on sleep quality. The results are shown in Table 5. Meanwhile, all three indirect pathways were verified significant: crisis perception → anxiety → sleep quality (effect = 0.142, 95%CI = [0.122, 0.161]), crisis perception → sleep procrastination → sleep quality (effect = 0.043, 95%CI = [0.034, 0.052]), and crisis perception → anxiety → sleep procrastination → sleep quality (effect = 0.013, 95%CI = [0.009, 0.018]). The total indirect effect was 0.198. The partial mediation model was supported, with crisis perception → anxiety → sleep quality as the primary mediating pathway, and indirect effects dominated the overall total effect Figure 2.

**Table 5:** The mediation effect analysis (*n* = 2,486).

Paths	Effect	95%CI
X1 → M1 → Y	0.142	0.122–0.161
X1 → M2 → Y	0.043	0.034–0.052
X1 → M1 → M2 → Y	0.013	0.009–0.018
Total indirect effect	0.198	–

X1 = crisis perception, M1 = anxiety, M2 = sleep procrastination, Y = sleep quality.

**Figure 3:**
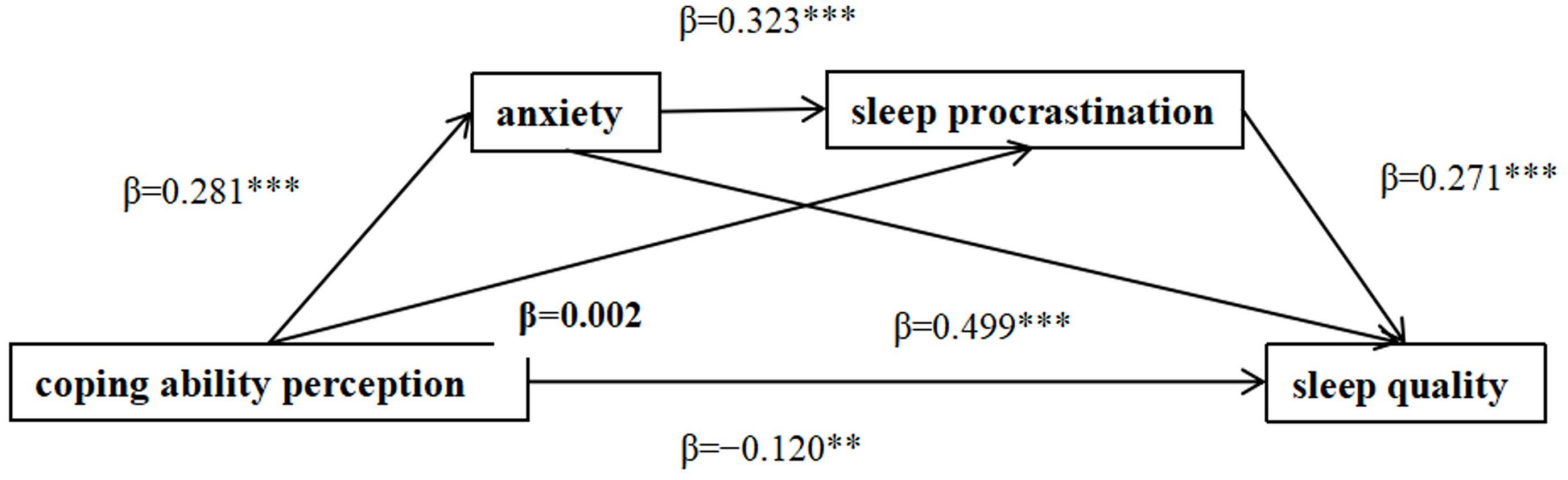
Diagram of the chain mediation model. ^***^p < 0.001, ^**^p < 0.01.

As can be seen from Table 6 and Figure 3. Bootstrap analysis with 5,000 resamples was conducted to examine the indirect effects, and distinct results were observed among three mediating pathways. First, the indirect pathway of coping ability perception → anxiety → sleep quality reached significance, with an effect size of 0.122 and 95%CI = [0.103, 0.143] (excluding zero). Second, the parallel independent pathway coping ability perception → sleep procrastination → sleep quality was non-significant, with an effect of 0.001 and 95%CI = [−0.008, 0.009] (containing zero). Third, the sequential chain pathway coping ability perception → anxiety → sleep procrastination → sleep quality showed significant indirect effect, with effect value of 0.022 and 95%CI = [0.017, 0.027]. The total indirect effect of coping ability perception on sleep quality was 0.145. Collectively, the indirect influence of coping ability perception on sleep quality was merely realized via the independent anxiety-mediated pathway and the sequential anxiety-sleep procrastination chain pathway. Combined with the significant negative direct effect, coping ability perception exerted a composite mechanism with coexistent negative direct influence and positive indirect mediating effects (Figure 3).

**Table 6:** The mediation effect analysis (*n* = 2,486).

Paths	Effect	95%CI
X2 → M1 → Y	0.122	0.103–0.143
X2 → M2 → Y	0.001	−0.008 – 0.009
X2 → M1 → M2 → Y	0.022	0.017–0.027
Total indirect effect	0.145	–

X2 = coping ability perception, M1 = anxiety, M2 = sleep procrastination, Y = sleep quality.

## 4 Discussion

The current study aimed to examine the link between perceived stress and sleep quality among graduate students, while identifying potential factors that influenced this relationship. Research has indicated that graduate education places substantial demands on specialized knowledge, research skills, innovative thinking, and academic output; furthermore, the training process is characterized by considerable challenges and uncertainties (Zheng et al., 2024; Bussell et al., 2020). Consequently, graduate students tend to endure high psychological stress, rendering them more vulnerable to sleep disturbances and anxiety symptoms (Townsend, 2010). Our findings revealed that 37.8% of Chinese university graduate students in this study experienced sleep disturbances. Prior work has documented the high prevalence of sleep disturbances in Chinese college students (25.7%, Li et al., 2018), consistent sleep problems among graduate students worldwide, and a global insomnia prevalence of 30–35% in adults (Hale et al., 2020). Collectively, the existing literature and our current results demonstrate that graduate students in Chinese universities are at increased risk of sleep disorders, highlighting the need for targeted psychological interventions. Furthermore, our study identified anxiety and sleep procrastination as key mediators of the relationship between perceived stress and sleep quality.

Our findings suggest that perceived stress has a direct effect on sleep quality. Past studies have reached similar conclusions indicating a direct negative association between perceived stress and sleep quality. When individuals are exposed to high levels of stress, their sleep quality tends to suffer, as evidenced by difficulties in falling asleep, light sleep, and increased nighttime awakenings (Eliasson et al., 2010; Choi et al., 2018; Herawati and Gayatri, 2019; Taylor et al., 2013; Van Reeth et al., 2000). These phenomena may have been related to the physiological and emotional responses induced by perceived stress. At the physiological level, perceived stress activates a stress response system, leading to changes in the endocrine system, such as an increase in corticotrophin-releasing factor, which affects sleep mechanisms (Heffner, 2024). Emotionally, perceived stress may trigger emotional problems such as anxiety, which further disrupts the sleep process.

Furthermore, this study examined the mediating role of anxiety in the relationship between perceived stress (as measured by the dimensions of crisis perception and coping ability perception) and sleep quality. This finding aligned with the conclusions of Johnson et al. (2006), who suggested that exposure to stress often leads to the development of anxiety, which manifests as nervousness, irritability, and fear. These emotional states further affect sleep (Johnson et al., 2006). On the one hand, anxiety may hinder sleep onset and increase nocturnal awakenings, thereby diminishing sleep quality. On the other hand, chronic anxiety may also affect circadian rhythms, further exacerbating sleep disturbances.

Our hypothesis predicted that sleep procrastination would mediate the relationship between perceived stress (on the dimension of crisis perception) and sleep quality. The present findings confirmed the mediating role of sleep procrastination in the association between perceived stress and sleep quality among Chinese university graduate students, supporting our hypothesis. These results are consistent with previous research (Ma et al., 2022). Sleep procrastination is characterized by delayed sleep onset and reduced sleep quality, and may be accompanied by symptoms such as drowsiness, fatigue, and impaired concentration. When individuals feel stressed, they may prefer to engage in delayed sleep as a means of avoidance, which feeds a self-reinforcing cycle: greater stress increases the likelihood sleep procrastination which in turn intensifies fatigue and perceived stress.

Considering the relative strength of the mediating pathways and potential alternative directions, our results consistently demonstrated that the mediating effect of anxiety was significantly stronger than that of sleep procrastination. This finding warrants deeper reflection, which we offer here. First, mechanistic differences may account for this disparity. Anxiety directly disrupts the physiological processes of sleep via somatic arousal (e.g., elevated cortisol, racing thoughts), whereas sleep procrastination primarily affects sleep through behavioral delay. The former exerts a more immediate and direct detrimental impact on sleep architecture (Gross and Levenson, 1995). Second, the unique contextual features of Chinese graduate education may amplify this difference. A meta-analysis of 330 studies involving 243,161 Chinese graduate students found that the prevalence of anxiety was 16.0% (Yu and Wang, 2024). Chinese master’s students face multiple sources of anxiety, primarily concentrated on thesis/dissertation completion, employment issues, and financial burden (Gao et al., 2024). A nationwide survey of master’s graduates in China revealed that over 60% of students in disciplines such as management, education, and history reported anxiety related to thesis/dissertation completion, while over 30% in economics, literature, and arts reported employment-related anxiety (Gao et al., 2024). Furthermore, under the pervasive ‘involution’ (neijuan) context—characterized by escalating competition with diminishing returns—master’s students in China are exposed to dual academic research pressures (Shang et al., 2025). Hindrance-type research pressure, in particular, was found to be significantly positively correlated with inhibitory anxiety (*r* = 0.56, *p* < 0.01), suggesting that when students appraise academic demands as insurmountable obstacles, anxiety is preferentially triggered (Shang et al., 2025). Doctoral students in China facing a high-stakes ‘publish or perish’ evaluation system also report high career anxiety (Horta and Li, 2022), and this pattern is increasingly mirrored among master’s students who are subject to similar publication expectations. These stressors are more likely to be appraised by individuals as ‘threats’ rather than ‘challenges,’ thereby preferentially triggering anxiety (an emotional response) rather than mere behavioral procrastination. Consequently, for this population, emotion regulation techniques targeting anxiety (e.g., mindfulness, cognitive reappraisal) may be more critical for improving sleep quality than purely behavioral management (e.g., time planning).

Furthermore, while our proposed model was supported, we acknowledge the possibility of reverse or bidirectional relationships. Poor sleep quality itself is a well-established risk factor for next-day perceived stress and heightened anxiety (Chellappa and Aeschbach, 2022). Longitudinal evidence indicates that poor sleep quality negatively predicts subsequent internalizing symptoms and vice versa, forming a bidirectional association (Meng et al., 2025). Notably, the same study also demonstrated that perceived stress mediates the impact of poor sleep quality on anxiety (Meng et al., 2025). Intensive daily-life studies also reveal that better-than-usual subjective sleep quality predicts lower next-day momentary stress (Bamert et al., 2025). Pre-existing poor sleepers are at more than twice the odds of developing generalized anxiety disorder under chronic stress (Kalmbach et al., 2018). Chronic sleep disturbances may lower the threshold for stress appraisal and emotional reactivity, creating a vicious cycle. Our cross-sectional design cannot disentangle these directions. Future longitudinal studies should test both the forward (stress →sleep) and reverse (sleep → stress → anxiety) pathways to fully capture the dynamic interplay among these variables.

Our findings further supported a significant chain mediation pathway: perceived stress may initially elicit anxiety, which subsequently promotes sleep procrastination behaviors, ultimately exerting a combined effect on sleep quality. This chain mediation effect draws attention to the substantial impact of perceived stress on sleep quality and underscores the crucial role of the mediation factors involved. A large body of prior research has consistently identified anxiety as a key contributor to pre-sleep procrastination (Liu et al., 2024). Specifically, when experiencing anxiety, individuals may seek temporarily distraction by engaging in activities that demand minimal cognitive effort and offer immediate gratification, such as scrolling through their phones or watching videos, rather than adhering to a regular sleep schedule (Peng et al., 2025). This behavior leads to intentional bedtime delay, ultimately resulting in significant sleep procrastination. Consequently, the sleep deprivation and diminished sleep quality associated with sleep procrastination can contribute to negative outcomes such as depression and anxiety (Huang et al., 2024). In addition, this study differentiates between two dimensions of perceived stress—crisis perception and coping ability perception—by examining how each exerts unique downstream effects on sleep quality via the serial mediators of anxiety and bedtime procrastination. Findings indicate that crisis perception primarily provokes acute state anxiety as a direct response to external threats. This state anxiety subsequently promotes sleep procrastination (e.g., using smartphones to avoid sleep), which in turn compromises sleep quality. In contrast, coping ability perception influences sleep quality through a more protracted, chronic pathway. In this pathway, low coping ability perception undermines an individual’s self-efficacy in regulating emotions. This deficit gives rise to a diffuse form of anxiety and fosters habitual sleep procrastination, which is driven by a self-defeating mindset of “I cannot control myself anyway.” Interventions should accordingly prioritize anxiety reduction for high-crisis individuals and efficacy-building for low-control individuals. In summary, the influence of perceived stress on sleep quality is a complex process, with anxiety and sleep procrastination serving as key mediating factors at the core of this process. Therefore, improving sleep quality requires not only addressing the stressors themselves, but also considering the influence of these mediating factors. By implementing strategies to alleviate anxiety and reduce sleep procrastination, the negative impact of perceived stress on sleep quality can be mitigated. This approach not only enhances physical and mental health but also promotes a healthier and more positive lifestyle.

Sleep has a significant impact on various aspects of students’ academic performance, emotional well-being, and mental health. Students with good sleep habits are better able to internalize what they have learned, thereby significantly improving their learning efficiency. In contrast, sleep deprivation directly disrupts memory consolidation, leading to a marked decline in learning outcomes (Haile et al., 2017). Furthermore, good sleep quality alleviates negative emotions such as stress, anxiety, and depression, and also helps enhance positive emotional experiences (Mathew et al., 2023; Selim et al., 2026). Thus, sufficient and high-quality sleep is a critical foundation for students to achieve their academic potential and improve learning outcomes, while also serving as a key factor maintaining physical and mental health and promoting emotional well-being.

Beyond its theoretical contributions, our study also has practical implications for graduate students and university administrators. During the postgraduate stage, students often face demanding academic workloads and intense research pressure, which can result in excessive perceived stress. Students need to learn how to manage their mindset and proactively cope with these pressures. Engaging in physical exercise and appropriate social interactions can alleviate stress and promote a positive outlook (Rassolnia and Nobari, 2024). In addition, schools and teachers should also closely monitor the psychological well-being of students, provide psychological counseling services, and offer necessary guidance and support. Anxiety is a common psychological issue among postgraduate students, often strongly correlated with academic and career-related pressures. To mitigate anxiety, students can be encouraged to adopt relaxation techniques, such as deep breathing and meditation (Jia et al., 2025). Furthermore, fostering healthy lifestyle habits is crucial for improving anxiety symptoms and sleep quality. Maintaining a regular sleep schedule, avoiding late-night activities, and limiting excessive use of electronic devices can help alleviate anxiety and improve sleep quality (Monk et al., 2011; Zeng et al., 2026). Sleep procrastination, which frequently results in insufficient and poor-quality sleep, requires targeted interventions. To address this issue, students need to recognize the importance of sleep and cultivate good sleep habits. In addition, educational institutions can implement measures to help students overcome sleep procrastination, such as monitoring students’ sleep patterns and establishing reasonable sleep guidelines.

## 5 Limitations

Despite its comprehensive scope, the current study has several limitations. First, due to the cross-sectional design, causal relationships among the variables could not be established, and some findings should be interpreted with caution. Future research employing longitudinal or experimental designs is needed to validate the conclusions drawn in this study. Second, this study focused solely on the relationships among perceived stress, anxiety, sleep procrastination and sleep quality, as well as differences in basic demographic variables. Other potential factors influencing sleep quality among graduate students in Chinese universities warrant further investigation. Third, the current study used self-report measures, making it susceptible to response biases.

## 6 Conclusion

The present study aimed to explore the relationship between perceived stress and sleep quality among Chinese university graduate students using a chain mediation model. The findings revealed that the prevalence of sleep disturbances among Chinese university graduate students was 37.8%. Furthermore, the results indicated that perceived stress was associated with increased anxiety, which subsequently led to increased sleep procrastination, ultimately contributing to impaired sleep quality.

## Data Availability

The datasets used and/or analyzed during the current study are available from the corresponding author on reasonable request.

## References

[ref9001] Allen H. K. Barrall A. L. Vincent K. B. Arria A. M. (2021). Stress and Burnout Among Graduate Students: Moderation by Sleep Duration and Quality. Int J Behav Med. 28, 21–28. doi: 10.1007/s12529-020-09867-8 32124246 PMC7483179

[ref1] Bamert M. Schwerdtfeger A. R. Rominger C. Inauen J. The relationship between stress and sleep quality: an intensive longitudinal study. [Epubh ahead of preprint]. (2025). 10.31234/osf.io/qfkyr_v1

[ref2] Bussell H. Schnabel J. Rinehart A. K. (2020). Meeting graduate student needs: an exploration of disciplinary differences. Public Serv. Q. 16, 213–233. doi: 10.1080/15228959.2020.1818663

[ref3] Chellappa S. L. Aeschbach D. (2022). Sleep and anxiety: from mechanisms to interventions. Sleep Med. Rev. 61:101583. doi: 10.1016/j.smrv.2021.101583, 34979437

[ref4] Choi D.-W. Chun S.-Y. Lee S. A. Han K.-T. Park E.-C. (2018). Association between sleep duration and perceived stress: salaried worker in circumstances of high workload. Int. J. Environ. Res. Public Health 15:796. doi: 10.3390/ijerph15040796, 29671770 PMC5923838

[ref9002] Cohen S. Williamson G. M. (1988). Perceived Stress in a Probability Sample of The United-States. 4th Annual Claremont Symp On Applied Social Psychology: Social Psychology Of Health. 31–67.

[ref5] Dong C. Xia L. Zhao C. Zhang X. He J. Zhang G. . (2023). Prospective association between perceived stress and anxiety among nursing college students: the moderating roles of career adaptability and professional commitment. BMC Psychiatry 23:388. doi: 10.1186/s12888-023-04887-6, 37264378 PMC10234240

[ref6] Eliasson A. H. Kashani M. Mayhew M. Ude A. Hoffman J. Vernalis M. . (2010). Reducing perceived stress improves sleep quality: a longitudinal outcomes study. Chest 138:913A. doi: 10.1378/chest.1041720472861

[ref7] Gao Y. Cao L. Z. Xu D. D. (2024). Sources and influencing factors of anxiety among professional master's students. Acad. Degrees Grad. Educ. 1, 49–56.

[ref8] Garbóczy S. Szemán-Nagy A. Ahmad M. S. Harsányi S. Ocsenás D. Rekenyi V. . (2021). Health anxiety, perceived stress, and coping styles in the shadow of the COVID-19. BMC Psychol. 9, 1–13. doi: 10.1186/s40359-021-00560-3, 33823945 PMC8022303

[ref9] Gross J. J. Levenson R. W. (1995). Emotional suppression: physiology, self-report, and expressive behavior. J. Pers. Soc. Psychol. 64, 970–986. 10.1037//0022-3514.64.6.970 8326473

[ref10] Haile Y. G. Alemu S. M. Habtewold T. D. (2017). Insomnia and its temporal association with academic performance among university students: a cross-sectional study. Biomed. Res. Int. 20, 1–7. doi: 10.1155/2017/2542367, 28752093 PMC5511682

[ref11] Hale L. Troxel W. Buysse D. J. (2020). Sleep health: an opportunity for public health to address health equity. Annu. Rev. Public Health 41, 81–99. doi: 10.1146/annurev-publhealth-040119-094412, 31900098 PMC7944938

[ref12] Harvey A. G. (2002). A cognitive model of insomnia. Behav. Res. Ther. 40, 869–893. doi: 10.1016/S0005-7967(01)00061-4, 12186352

[ref13] Heffner J. S. (2024). Enhancing sleep quality for adult patients: interventions and insights. Nurse Pract. 49, 30–31. doi: 10.1097/01.NPR.0000000000000208 38915147

[ref14] Herawati K. Gayatri D. (2019). The correlation between sleep quality and levels of stress among students in Universitas Indonesia. Enferm. Clin. 29, 357–361. doi: 10.1016/j.enfcli.2019.04.044

[ref15] Hill V. M. Rebar A. L. Ferguson S. A. Shriane A. E. Vincent G. E. (2022). Go to bed! A systematic review and meta-analysis of bedtime procrastination correlates and sleep outcomes. Sleep Med. Rev. 66:101697. doi: 10.1016/j.smrv.2022.101697, 36375334

[ref16] Horta H. Li H. (2022). Nothing but publishing: the overriding goal of PhD students in mainland China, Hong Kong, and Macau. Stud. High. Educ. 48, 263–282. doi: 10.1080/03075079.2022.2131764, 37339054

[ref17] Huang Y. Zhang L. Li N. Zhihua X. Yan D. (2024). The relationship between social media addiction and anxiety among college students: the mediating role of bedtime procrastination and the moderating role of perceived stress. Mod. Prev. Med. 51, 3756–3761. doi: 10.20043/j.cnki.MPM.202407048

[ref18] Jia Y. Qian T. Wang R. Yu X. Ma Y. (2025). Effects of mindfulness meditation training on negative emotions and sleep quality in patients with depression. J. Clin. Psychosom. Dis. 31, 29–32.

[ref19] Johnson E. O. Roth T. Breslau N. (2006). The association of insomnia with anxiety disorders and depression: exploration of the direction of risk. J. Psychiatr. Res. 40, 700–708. doi: 10.1016/j.jpsychires.2006.07.008, 16978649

[ref20] Kalmbach D. A. Fang Y. Drake C. L. . (2018). Insomnia symptoms and short sleep predict anxiety and worry in response to stress exposure: a prospective cohort study of medical interns. Sleep Med. 55, 60–67. 10.1016/j.sleep.2018.12.001 PMC7045299 30763868

[ref21] Kernan W. Bogart J. Wheat M. E. (2011). Health-related barriers to learning among graduate students. Health Educ. 111, 425–445. doi: 10.1108/09654281111161248

[ref22] Kroese F. M. De Ridder D. T. Evers C. Adriaanse M. A. (2014). Bedtime procrastination: introducing a new area of procrastination. Front. Psychol. 5:89333. doi: 10.3389/fpsyg.2014.00611, 24994989 PMC4062817

[ref23] Lee H. Rauktis M. E. Fusco R. A. (2022). Perceived stress and sleep quality among master's students in social work. Soc. Work. Educ. 41, 1018–1034. doi: 10.1080/02615479.2021.1910231

[ref24] Li L. Wang Y. Y. Wang S. B. Zhang L. Li L. Xu D. D. . (2018). Prevalence of sleep disturbances in Chinese university students: a comprehensive meta-analysis. J. Sleep Res. 27:e12648. doi: 10.1111/jsr.12648, 29383787

[ref26] Liu N. Wang J. Zang W. (2024). The impact of sleep determination on procrastination before bedtime: the role of anxiety. Int. J. Ment. Health Promot. 26, 377–387.

[ref9003] Liu X. C. Tang M. Q. Hu L. Wang A. Z. Wu H. X. Zhao G. F. . (1996). A study on the reliability and validity of the Pittsburgh sleep quality index. Chin. J. Psychiatry. 29:107.

[ref25] Li X. Wei X. Chen H. Gao L. Li W. (2019). Relationship between perceived stress and perceived sleep quality: a dual-stage moderated mediation model among university students. Chin. J. Clin. Psychol. 2, 351–355.

[ref28] Mathew G. M. Reichenberger D. A. Master L. Buxton O. M. Chang A.-M. Hale L. (2023). Actigraphic sleep variability is associated with lower positive mood in adolescents. J. Adolesc. Health 73, 478–485. doi: 10.1016/j.jadohealth.2023.04.019, 37410005 PMC10524712

[ref27] Ma X. Meng D. Zhu L. Xu H. Guo J. Yang L. . (2022). Bedtime procrastination predicts the prevalence and severity of poor sleep quality of Chinese undergraduate students. J. Am. Coll. Heal. 70, 1104–1111. doi: 10.1080/07448481.2020.1785474, 32669056

[ref29] Meng R. Xu J. Luo Y. Mastrotheodoros S. Jiang C. Garofalo C. . (2025). Perceived stress mediates the longitudinal effect of sleep quality on internalizing symptoms. J. Affect. Disord. 373, 51–59. doi: 10.1016/j.jad.2024.12.046, 39675679

[ref30] Monk T. H. Buysse D. J. Billy B. D. Fletcher M. E. Kennedy K. S. Schlarb J. E. . (2011). Circadian type and bed-timing regularity in 654 retired seniors: correlations with subjective sleep measures. Sleep 34, 235–239. doi: 10.1093/sleep/34.2.235, 21286245 PMC3022945

[ref31] Muraven M. Baumeister R. F. (2000). Self-regulation and depletion of limited resources: does self-control resemble a muscle? Psychol. Bull. 126, 247–259. doi: 10.1037/0033-2909.126.2.247, 10748642

[ref32] Oh C.-M. Kim H. Y. Na H. K. Cho K. H. (2019). The effect of anxiety and depression on sleep quality of individuals with high risk for insomnia: a population-based study. Front. Neurol. 10:477232. doi: 10.3389/fneur.2019.00849 PMC6700255 31456736

[ref33] Peng J. Zhang M. Xiao J. Li K. Zhao Y. Li Y. (2025). Cross-sectional analysis of the relationship between bedtime procrastination and fear of missing out and the effect of exercise intervention. Acta Acad. Med. Sin. 47, 175–181. doi: 10.3881/j.issn.1000-503X.16275 40331375

[ref34] Rassolnia A. Nobari H. (2024). The impact of socio-economic status and physical activity on psychological well-being and sleep quality among college students during the COVID-19 pandemic. Int. J. Sport. Stud. Health 7, 1–12. doi: 10.61838/kman.intjssh.7.2.1

[ref35] Selim M. Y. Shaaban S. F. Shoma N. A. ElLeithi A. A. Shahin R. Y. Ibrahim I. M. A. (2026). A cross-sectional study of sleep quality and its association with academic performance and psychological distress among medical students in Mansoura University, Egypt. Egypt. J. Neurol. Psychiatr. Neurosurg. 62, 5–5. doi: 10.1186/s41983-026-01070-y

[ref36] Shang H. Gao T. Niu L. (2025). Study on the mechanism of dual academic research pressure on anxiety among master's students under an involution context: evidence from a survey of 46 Chinese universities. Front. Psychol. 16:1667922. doi: 10.3389/fpsyg.2025.1667922, 41346512 PMC12672908

[ref37] Taylor N. D. Fireman G. D. Levin R. (2013). Trait hostility, perceived stress, and sleep quality in a sample of normal sleepers. Sleep Disord. 2013:735812. doi: 10.1155/2013/735812 23766918 PMC3655654

[ref38] Townsend J. C. (2010). The Moderating Role of Social Support on the Relationship of Perceived Stress and Life Satisfaction of Psychology Graduate Students. Lawrence, Kansas, USA: University of Kansas.

[ref39] Van Reeth O. Weibel L. Spiegel K. Leproult R. Dugovic C. Maccari S. (2000). Physiology of sleep (review)--interactions between stress and sleep: from basic research to clinical situations. Sleep Med. Rev. 4, 201–219. doi: 10.1053/smrv.1999.0097

[ref40] Wyatt T. Oswalt S. B. (2013). Comparing mental health issues among undergraduate and graduate students. Am. J. Health Educ. 44, 96–107. doi: 10.1080/19325037.2013.764248

[ref43] Yuan L. Lin N. (2009). A study on the factor structure of the perceived stress scale in a sample of university students. J. Guangdong Second Normal Univ. 29, 45–49.

[ref42] Yu G. L. Wang X. Z. (2024). Basic conditions and educational countermeasures of mental health problems in Chinese graduate students. China High. Educ. Res. 40, 880–887. doi: 10.16298/j.cnki.1004-3667.2024.07.12

[ref44] Zeng L. Lu S. Zhao Y. M. Li W. He Y. J. Liu Y. (2026). Evaluation of the impact of mobile phone addiction and anxiety-depression levels on sleep quality among college students based on a decision tree model. Int. J. Psychiatry 1, 126–130. doi: 10.13479/j.cnki.jip.2026.01.028

[ref45] Zheng Z. Lin M. Yue H. (2024). An exploration of postgraduate students’ innovative capabilities: a review and outlook. Res. Contin. Educ. 7, 101–106.

[ref46] Zung W. W. K. (1971). A rating instrument for anxiety disorders. Psychosomatics 12, 371–379. 5172928 10.1016/S0033-3182(71)71479-0

